# Supplementary material for: Usnic Acid Derivatives Inhibit DNA Repair Enzymes Tyrosyl-DNA Phosphodiesterases 1 and 2 and Act as Potential Anticancer Agents
Source: Genes (Basel). 2023 Oct 12;14(10):1931. doi: 10.3390/genes14101931 (PMC10606488; doi:10.3390/genes14101931)
Supplement: Supplementary file 1 [file genes-14-01931-s001.zip › genes-2567336-supplementary.pdf]

Supplementary

Usnic acid derivatives inhibit DNA repair enzymes Tyrosyl-DNA phosphodiesterases 1 and 2 and act as potential anticancer agents

Alexandra L. Zakharenko [1], Nadezhda S. Dyrkheeva [1], Olga A. Luzina [2], Aleksandr S. Filimonov [2], Evgenii S. Mozhaitsev [2], Anastasia A. Malakhova [3], Sergey P. Medvedev [3], Suren M. Zakian [3], Nariman F. Salakhutdinov [2], and Olga I. Lavrik [1,*]

[1] Institute of Chemical Biology and Fundamental Medicine, Siberian Branch of the Russian Academy of Sciences, 8, Akademika Lavrentieva Ave., 630090 Novosibirsk, Russia; a.zakharenko73@gmail.com (A.L.Z.); dyrkheeva.n.s@gmail.com (N.S.D.); lavrik@niboch.nsc.ru (O.I.L.)

[2] N. N. Vorozhtsov Novosibirsk Institute of Organic Chemistry, Siberian Branch of the Russian Academy of Sciences, 9, Akademika Lavrentieva Ave., 630090 Novosibirsk, Russia; luzina@nioch.nsc.ru (O.A.L.); alfil@nioch.nsc.ru (A.S.F.); mozh@nioch.nsc.ru (E.S.M.); anvar@nioch.nsc.ru (N.F.S.)

[3] Federal Research Center Institute of Cytology and Genetics, Siberian Branch of the Russian Academy of Sciences, 10, Akademika Lavrentieva Ave., 630090 Novosibirsk, Russia; amal@bionet.nsc.ru (A.A.M.); medvedev@bionet.nsc.ru (S.P.M.); zakian@bionet.nsc.ru (S.M.Z.)

* Correspondence: lavrik@niboch.nsc.ru; Tel.: + 7-383-3635195

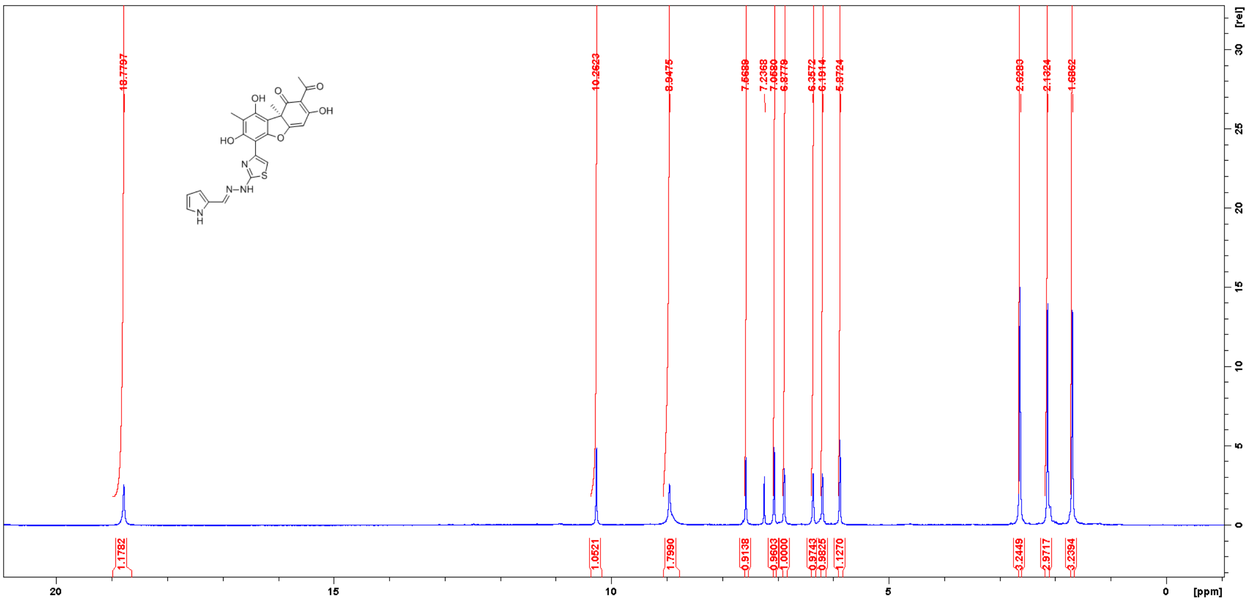

Figure S1. The ^{1}H spectrum of compound **11a**.

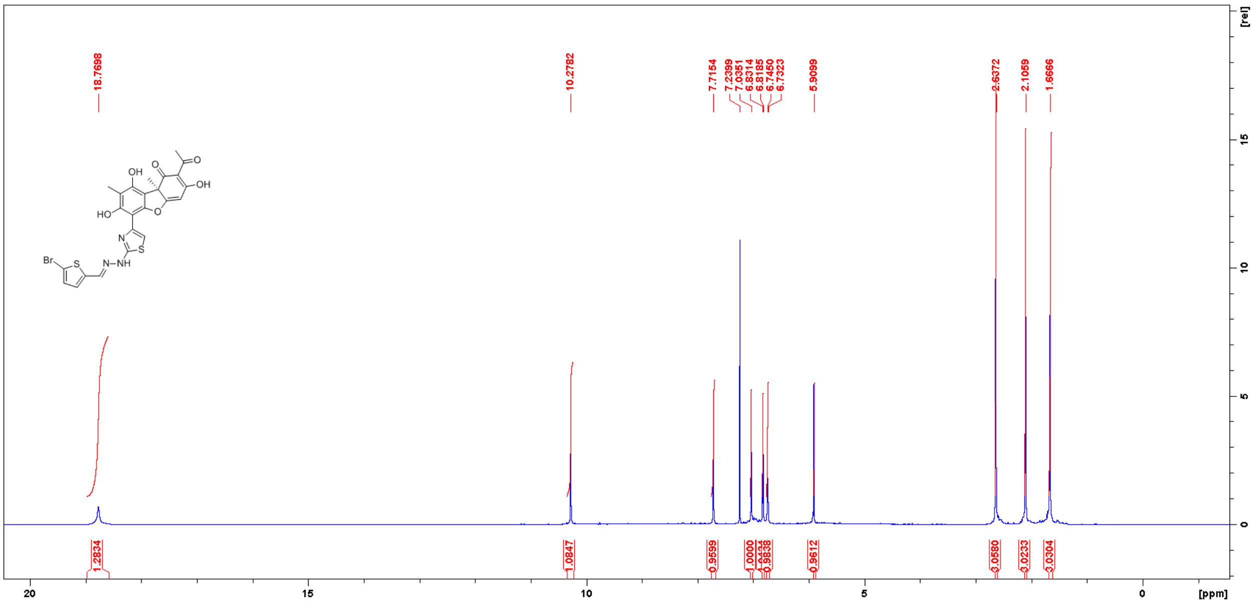

Figure S2. The ^{1}H spectrum of compound **11b**.

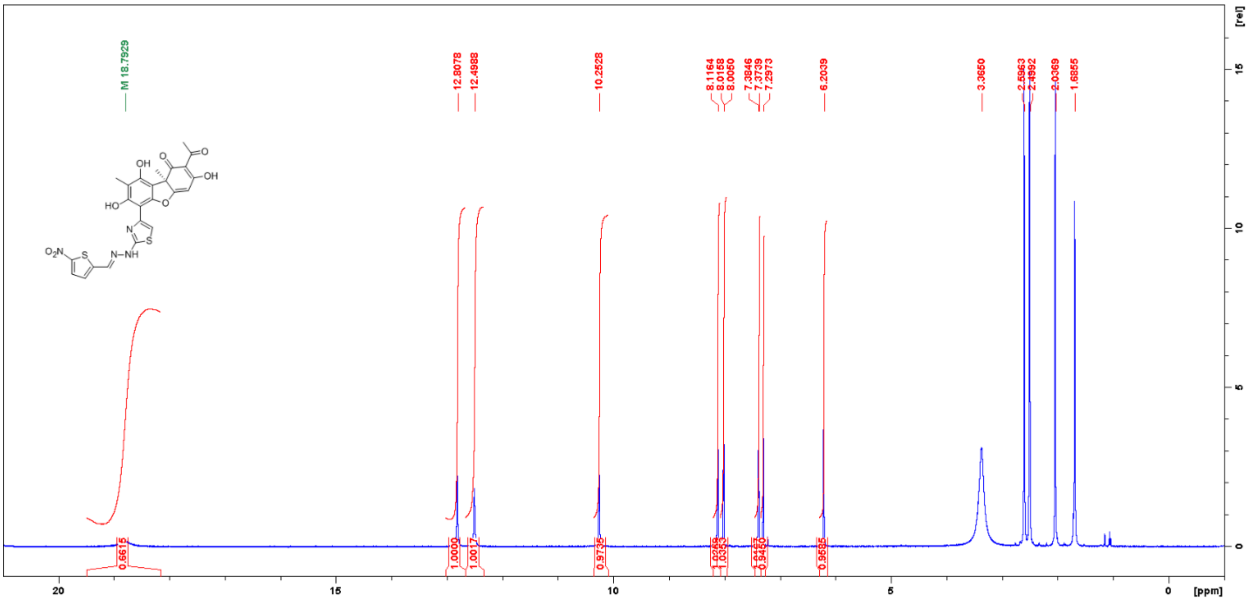

Figure S3. The ^{1}H spectrum of compound **11c**.

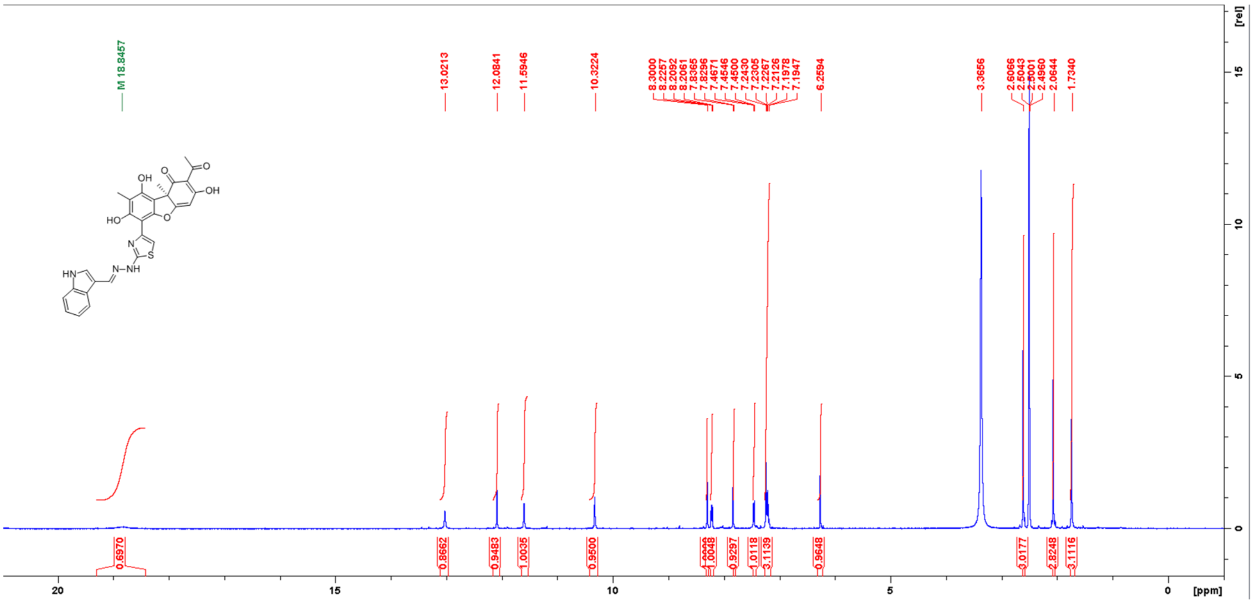

Figure S4. The ^{1}H spectrum of compound **11d**.

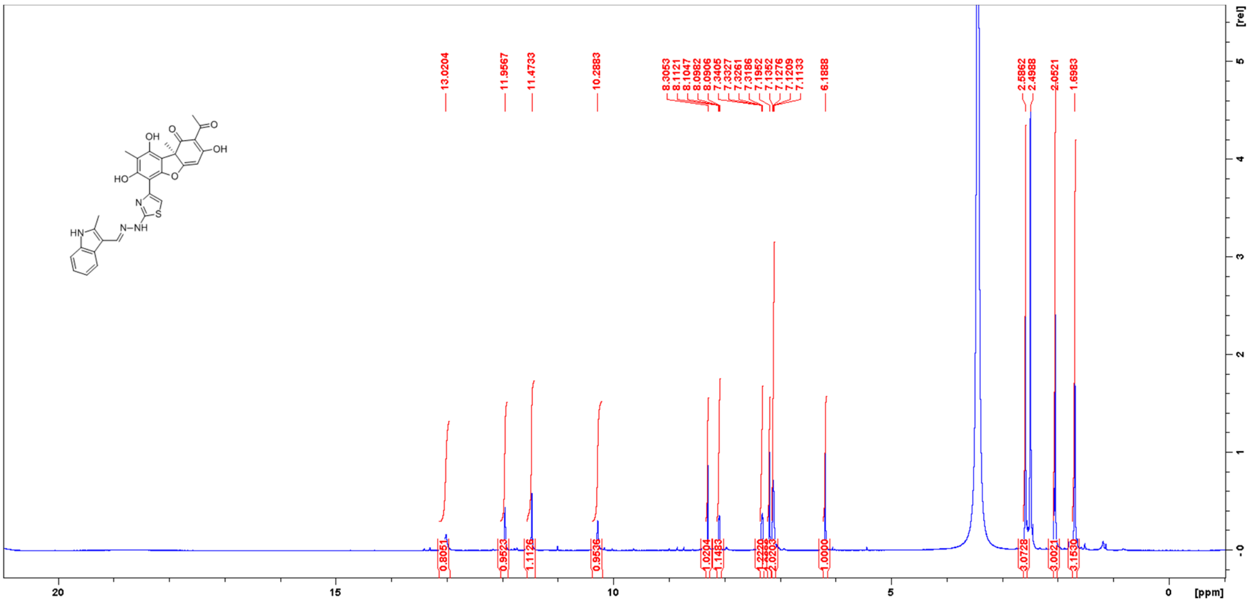

Figure S5. The ^{1}H spectrum of compound **11e**.

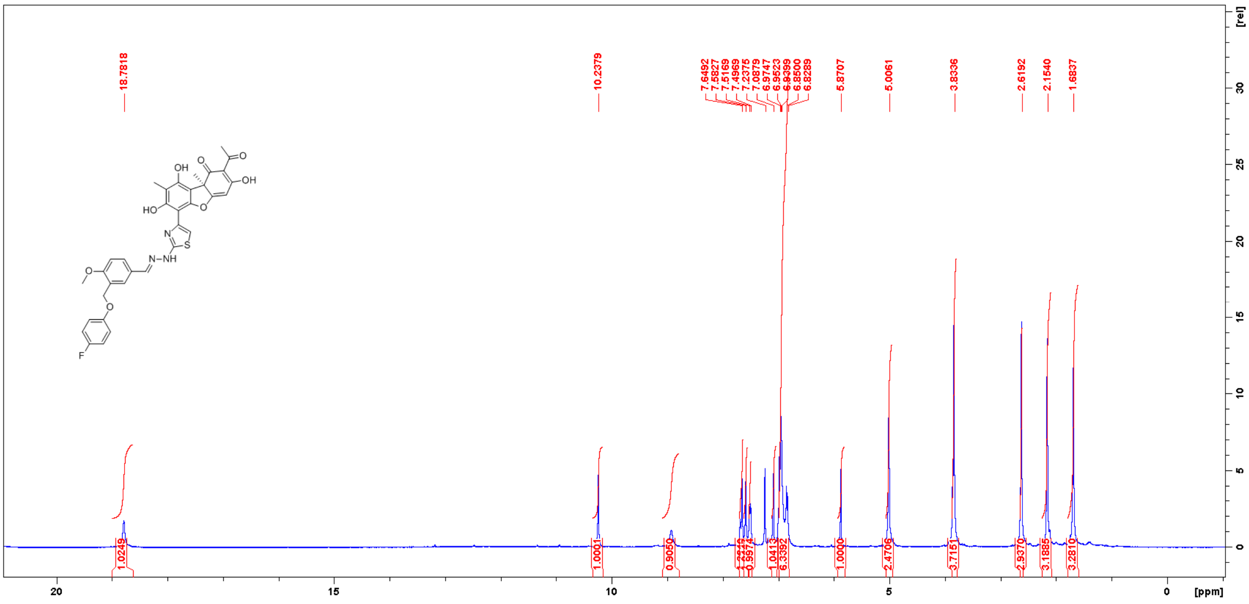

Figure S6. The ^{1}H spectrum of compound **11f**.

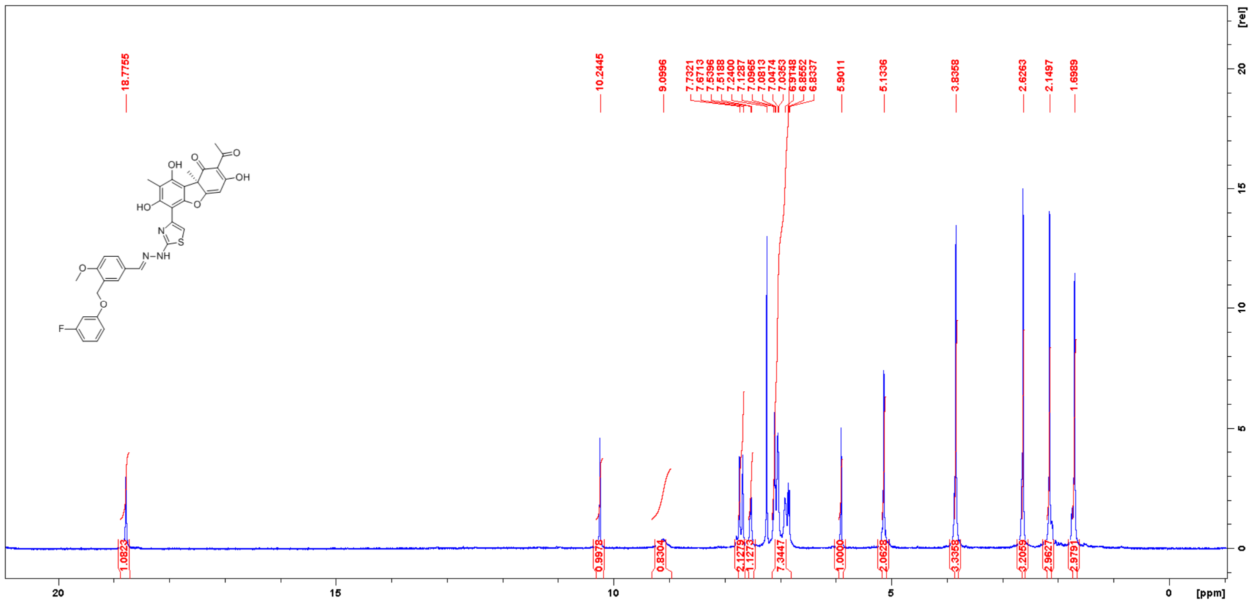

Figure S7. The ^{1}H spectrum of compound **11g**.

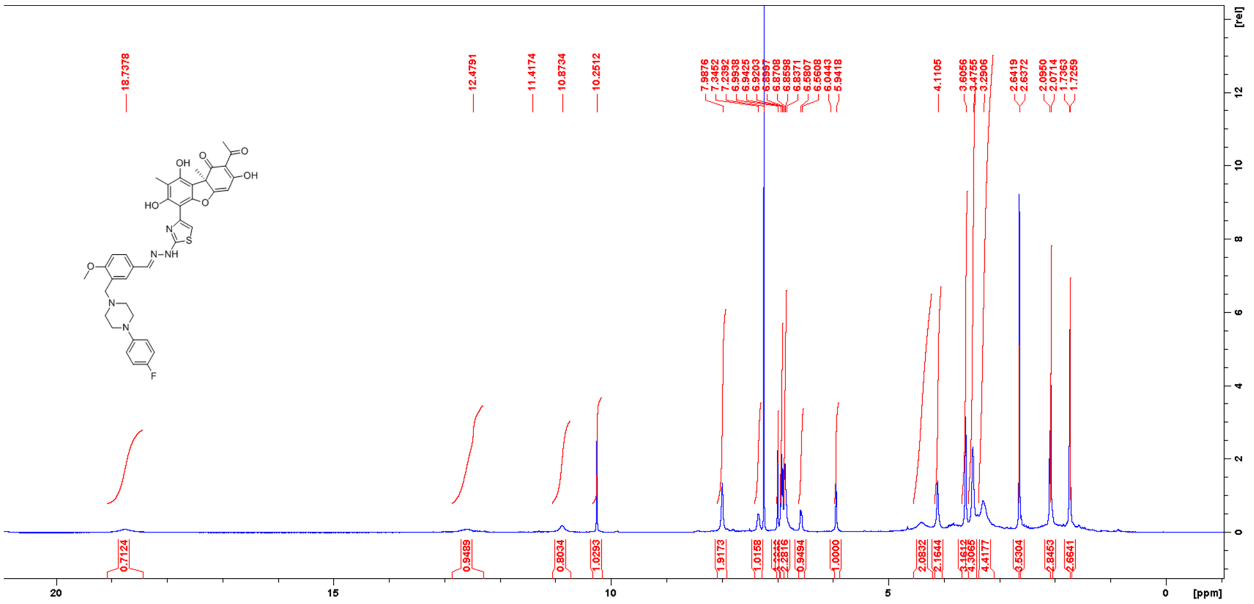

Figure S8. The ^{1}H spectrum of compound **11h**.

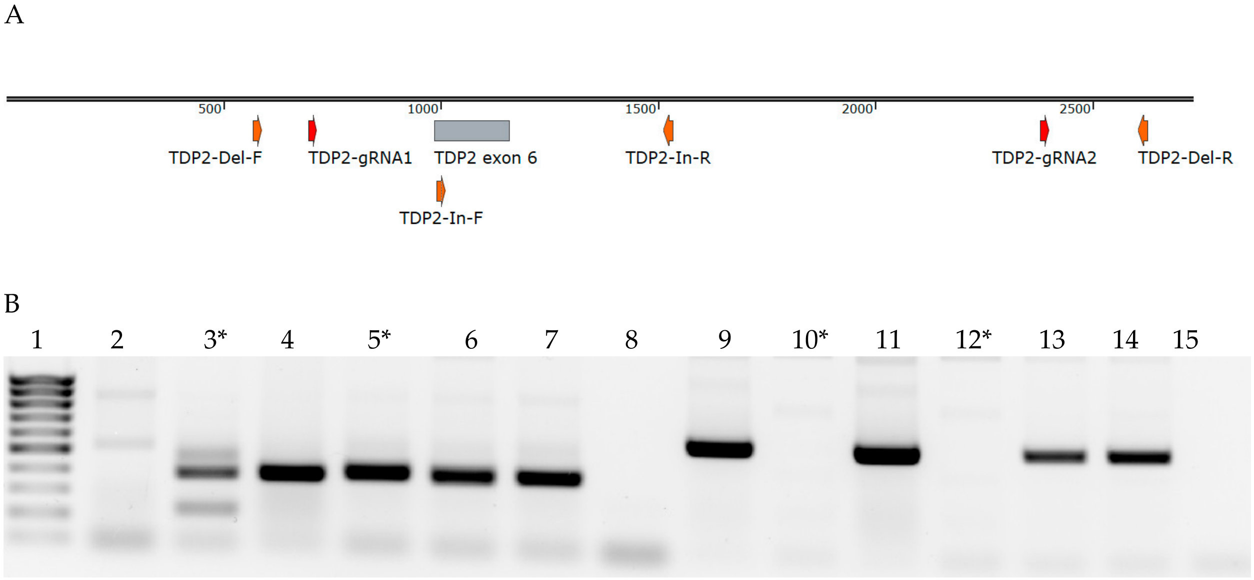

Figure S9. Scheme of protospacers (TDP2-gRNA1 and TDP2-gRNA1) and primers location in *TDP2* gene (A) and PCR analysis of CRISPR/Cas9-induced deletion (B): lanes: 1) DNA ladder Step100 (Biolabmix, Novosibirsk, Russia; 2-7) PCR analysis of HEK293FT clones genomic DNA with primers for detecting a deletions (TDP2-Del-F and TDP2-Del-R); 8) H_2O with primers TDP2-Del-F and TDP2-Del-R; 9-14) PCR analysis of HEK293FT clones genomic DNA with primers for the detection of the wild-type alleles (TDP2-In-F and TDP2-In-R); 15) H_2O with primers TDP2-In-F and TDP2-In-R; * - these two cell clones were selected for further analysis.

Table S1. Molecular docking results

ID ligand	Docking score	Emodel	IFD score	Interactions	
				π- π Stacking	Other interactions
**(-)-11a**	-8.463	-93.324	-469.56	Trp307, Tyr321	Arg276 π-cation<br>Ser236, Arg276, Asn274 - H-bond<br>Ley134, Trp307, Ley315, Tyr321 - hydrophobic
**(-)-11b**	-7.112	-94.008	-469.67	Phe325	Asn274 – H-bond<br>Ley315, Tyr321 - hydrophobic
**(-)-11c**	-8.033	-108.527	-469.14	Trp307	Arg276 π-cation<br>Arg276 - H-bond<br>Trp307, Tyr321 - hydrophobic
**(-)-11d**	-8.613	-113.831	-468.70	Trp307, Tyr321, His323	Arg241, Arg276 π-cation<br>Asn274, Arg276, Asp277, Lys322 - H-bond<br>Ley134, Trp307, Ley315, Tyr321 - hydrophobic
**(-)-11e**	-7.524	-98.671	-467.97	Trp307, Tyr321, His323	Arg241, Arg276 π-cation<br>Arg276, Asn274 - H-bond<br>Trp307, Ley315 - hydrophobic
**(-)-11g**	-7.335	-104.451	-468.41	Trp307, Tyr321	Arg276 π-cation<br>Ley315 - hydrophobic
**(-)-11h**	-8.649	-120.321	-468.01	Trp307, Phe325	Arg241 π-cation<br>Asn274, Arg276 - H-bond<br>Ley134, Trp307, Ley315 - hydrophobic
